# Identifying prognostic characteristics of m6A-related glycolysis gene and predicting the immune infiltration landscape in bladder cancer

**DOI:** 10.1186/s12935-023-03160-w

**Published:** 2023-11-28

**Authors:** Guanwen Zhou, Yi Li, Xiangguo Ren, Guoliang Qin, Zhaocun Zhang, Haifeng Zhao, Lijian Gao, Xianzhou Jiang

**Affiliations:** 1 Department of Urology, Qilu Hospital of Shandong University, Jinan, 250012 China; 2 Department of Urology, Qilu Hospital of Shandong University Dezhou Hospital, Dezhou, 253000 China

**Keywords:** Bladder urothelial carcinoma, m6A, Glucose metabolism, Prognosis, Biomarkers, Immune

## Abstract

**Backgrounds:**

Glucose metabolism is associated with the development of cancers, and m6A RNA methylation regulator-related genes play vital roles in bladder urothelial carcinoma (BLCA). However, the role of m6A-related glucose metabolism genes in BLCA occurrence and development has not yet been reported. Our study aims to integrate m6A- and glycolysis-related genes and find potential gene targets for clinical diagnosis and prognosis of BLCA patients.

**Methods:**

Sequencing data and clinical information on BLCA were extracted from common databases. Univariate Cox analysis was used to screen prognosis-related m6A glucose metabolism genes; BLCA subtypes were distinguished using consensus clustering analysis. Subsequently, genes associated with BLCA occurrence and development were identified using the “limma” R package. The risk score was then calculated, and a nomogram was constructed to predict survival rate of BLCA patients. Functional and immune microenvironment analyses were performed to explore potential functions and mechanisms of the different risk groups.

**Results:**

Based on 70 prognosis-related m6A glucose metabolism genes, BLCA was classified into two subtypes, and 34 genes associated with its occurrence and development were identified. Enrichment analysis revealed an association of genes in high-risk groups with tricarboxylic acid cycle function and glycolysis. Moreover, significantly higher levels of seven immune checkpoints, 14 immune checkpoint inhibitors, and 32 immune factors were found in high-risk score groups.

**Conclusions:**

This study identified two biomarkers associated with BLCA prognosis; these findings may deepen our understanding of the role of m6A-related glucose metabolism genes in BLCA development. We constructed a m6A-related glucose metabolism- and immune-related gene risk model, which could effectively predict patient prognosis and immunotherapy response and guide individualized immunotherapy.

**Supplementary Information:**

The online version contains supplementary material available at 10.1186/s12935-023-03160-w.

## Background

Bladder urothelial carcinoma (BLCA) is the most prevalent malignancy of the urinary system, with an estimated 573,000 new cases and 213,000 deaths in 2020, and is ranked as the 10th most common malignancy worldwide [1]. BLCA occurrence and development is a complex pathophysiological process, involving factors such as genetics and external environment. Currently recognized risk factors for BLCA include smoking and long-term exposure to chemicals [2, 3]. Although the development of treatment strategies for BLCA has rapidly advanced in recent years, the carcinoma is still characterized by a high recurrence rate and poor prognosis. Therefore, studying the molecular mechanisms underlying BLCA is particularly important to identifying new therapeutic targets.

N6-methyladenine (m6A) is the most common modification in eukaryotic cells at the mRNA and non-coding RNA levels [4]. m6A modification is a reversible process catalyzed by methyltransferases (writers), demethylases (erasers), and m6A-binding proteins (readers), and its aberrant regulation is associated with the development of multiple cancers [5, 6]. Reprogramming of energy metabolism is a hallmark of malignant tumors. The most prominent feature of this process is aerobic glycolysis, also known as the Warburg effect, where cancer cells rely on glycolysis for energy even when sufficient oxygen is present [7]. In addition to providing energy for tumor proliferation, this process generates metabolites that reshape the tumor microenvironment (TME) [8]. Therefore, targeting reprogrammed metabolic pathways is a potentially valuable therapeutic strategy. The m6A modification is involved in regulating expression of various glycolysis-related genes; however, the role of m6A-related glucose metabolism genes in BLCA occurrence and development has not been reported.

In this study, we integrated m6A- and glycolysis-related genes, and analyzed transcriptome and clinical data of BLCA from The Cancer Genome Atlas (TCGA) and Gene Expression Omnibus (GEO) databases. Based on the expression of m6A-related glycolytic genes associated with BLCA prognosis, BLCA cases were divided into subtypes. We identified critical differential genes related to BLCA occurrence and development, and constructed a prognostic model. Following this, we conducted independent prognostic and clinical correlation analyses of the model, and functional and immune microenvironment analyses of high- and low-risk groups. This study provides potential gene targets for clinical diagnosis and prognosis of BLCA patients.

## Methods

### Data extraction

RNA sequencing data, survival information, and clinical information of BLCA were downloaded from TCGA (https://portal.gdc.cancer.gov) and GEO databases (https://www.ncbi.nlm.nih.gov/geo/). The TCGA dataset, containing 411 BLCA samples and 19 normal control (HC) samples, was used as the training dataset. Within the dataset, 406 BLCA samples had both survival and clinical information. GSE32894, containing 308 BLCA samples of which 224 had both survival and clinical information, was used as the validation dataset to verify the availability of the prognostic model. We obtained a total of 753 glucose metabolism-related genes from the Molecular Signatures Database (MSigDB; https://www.gsea-msigdb.org/gsea/msigdb/), and 24 m6A RNA methylation regulator-related genes from the database of functional variants involved in RNA modification (RMVar, http://rmvar.renlab.org/index.html) (Additional file 1: Table S1) [9–11].

### Screening of prognosis-related m6A glucose metabolism genes

A Spearman correlation was performed between m6A methylation regulatory-related and glucose metabolism-related genes, to obtain m6A-related glycolytic genes with |Cor|> 0.5 and p < 0.05. A univariate Cox analysis was constructed to determine prognostic value of these genes and identify prognosis-related m6A glucose metabolism genes (p < 0.05).

### Survival and clinical characteristics analysis of different subtypes patients

Consensus clustering analysis is commonly used to divide samples into subtypes. The “ConsensusClusterPlus” R package (version 1.58.0) was used to analyze BLCA based on prognosis-related m6A glucose metabolism gene expression [12]. The best clustering method was selected based on cumulative distribution function values, and BLCA was divided into subtypes verified by principal component analysis.

Using the “survival” R package, Kaplan–Meier (KM) survival analysis was performed to study differences in survival rate between subtypes of patients (version 3.2–13) [13]. Clinical information (age, gender, overall survival, pathologic T, N, M, tumor stage) was collected. The chi-square test was used to select clinical characteristics of different subtypes, which were visualized using the “ComplexHeatmap” R package (version 1.14.0) [14].

### Screening of genes associated with BLCA occurrence and development

mRNA expression levels between BLCA and HC samples, as well as between patient subtypes, were compared using the “limma” R package (version 3.50.3) (adj. p-value < 0.05 and |log_2_fold change|> 0.5) [15]. Following this, genes associated with BLCA occurrence and development (target genes) were screened by using the online tool “Venny 2.1.0” to intersect the two groups of differentially expressed genes. In addition, Gene Ontology (GO) and Kyoto Encyclopedia of Genes and Genomes (KEGG) enrichment analyses were conducted using the “clusterprofiler” R package to investigate the functions of target genes (version 4.2.2) (adj.p-value < 0.05) [16].

### Construction of the prognostic model of BLCA

Biomarkers of BLCA obtained by Cox regression analysis were screened to construct a survival risk model. The risk score was calculated using the following algorithm, and high- and low-risk groups were delineated according to the median risk value.

$${\mathrm{Riskscore}}_{\mathrm{sample}}={\sum }_{\mathrm{n}=1}^{\mathrm{n}}{(\mathrm{Coef}}_{\mathrm{i}}*{\mathrm{x}}_{\mathrm{i}})$$

A KM survival analysis was performed between the two groups, and the accuracy of the survival risk model was predicted using the risk and receiver operating characteristic (ROC) curves. The GSE32894 dataset was used to verify the applicability of our model.

### Independent prognostic and clinical correlation analyses

The risk scores between different subtypes of clinical characteristics (age, gender, tumor stage, pathologic T, pathologic N, and pathologic M) were compared using the “wilcox.test” function. Then, univariate and multivariate Cox analyses were employed to identify the independent predictors for BLCA. The nomogram was constructed based on the independent predictors to predict the 1-, 3-, and 5-years survival rate. Its validity was then verified by drawing a calibration curve as well as a decision curve (DCA) at 1, 3, and 5 years of the prediction model.

### Analysis of function and immune microenvironment

Gene set enrichment analysis (GSEA) was used to explore functions of the high- and low-risk groups, and a ridge map was drawn based on the top five enrichment pathways.

The proportions of 22 immune cell types and functions in the high- and low-risk groups were calculated, and “wilcox.test” was used to compare the differences. Following this, Spearman’s correlations were performed between biomarkers and differentially immune cells, biomarkers and differentially immune functions, and risk score and differentially immune cells. The inter-group differences between the eight immune checkpoints, 24 immune checkpoint inhibitors, and 38 immune factors were calculated and compared using “wilcox.test” [17, 18]. Furthermore, immune phenotype scores (IPS) in different risk groups were calculated, and a Spearman’s correlation was performed to determine the relationship between IPS and risk score.

### Drug sensitivity analysis

The sensitivity of 138 commonly used drugs between high-risk and low-risk patient groups was analyzed and compared using the “pRRophetic” package (version 0.5) [19] and the “wilcox.test”, respectively. Drug sensitivity is measured by the half-maximal inhibitory concentration (IC50) value, with lower IC50 value indicating higher sensitivity of cells to the drug.

### Patient samples

Bladder cancer and paracancerous tissues were obtained from BLCA patients admitted to the Qilu Hospital of Shandong University, between 2021 and 2022. All participants were informed of the study and provided consent before undergoing surgery. This study was approved by the Institutional Review Board of the Qilu Hospital of Shandong University (No. 2020046).

### Cell culture

Bladder cancer cell lines 5637 and UM-UC-3 were purchased from the Type Culture Collection of the Chinese Academy of Sciences (Shanghai, China). All cell lines were tested for mycoplasma and resulted negative. 5637 cells were cultured in RPMI 1640 medium (Gibco, USA) supplemented with 10% fetal bovine serum (Gibco, USA). UM-UC-3 cells were cultured in Dulbecco’s modified medium (Gibco, USA) supplemented with 10% FBS (Gibco, USA). All cell lines were cultured in a 5% CO2 incubator at 37 °C.

### siRNA transfection

Cells were plated in six-well dishes and transfected with siRNA-PLA2G2F, siRNA-IP6K2, or negative control using Lipofectamine 3000 (Invitrogen, USA). All siRNA sequences are listed in Additional file 2: Table S2.

### RNA isolation and quantitative reverse transcription polymerase chain reaction (RT-qPCR)

Total RNA was extracted from tissues and cell lines using TRIzol reagent (Invitrogen, USA) and used to synthesize cDNA using Evo M-MLV RT Premix (Accurate Biology, China). RT-qPCR was performed using Premix Pro Taq HS qPCR Kit (Accurate Biology, China) on a LightCycler 96 (Roche, USA). β-actin was used as an internal control. All assays were performed in triplicate, and data were analyzed using the 2^−ΔΔCT^ method. All PCR primers were purchased from Accurate Biology (Shanghai, China), and are listed in Additional file 3: Table S3.

### Cell counting kit-8 (CCK-8) and colony formation assay

Cells were seeded in 96-well plates, with approximately 2000 cells per well. The Cell Counting kit-8 (CCK-8) (Bioss, China) was used to detect cell proliferation 0, 24, 48, 72, and 96 h after seeding. Absorbance was measured at 450 nm using a spectrophotometer (Tecan, Switzerland).

For colony formation assay, cells were seeded into 6-well plates with approximately 800 cells per well, and incubated at 5% CO2 and 37 °C. Colonies were fixed with 100% methanol and stained with crystal violet. Colonies containing more than 50 cells were considered survivors.

## Results

### Survival differences existed between patients in cluster 1 and 2

**Fig. 1:**
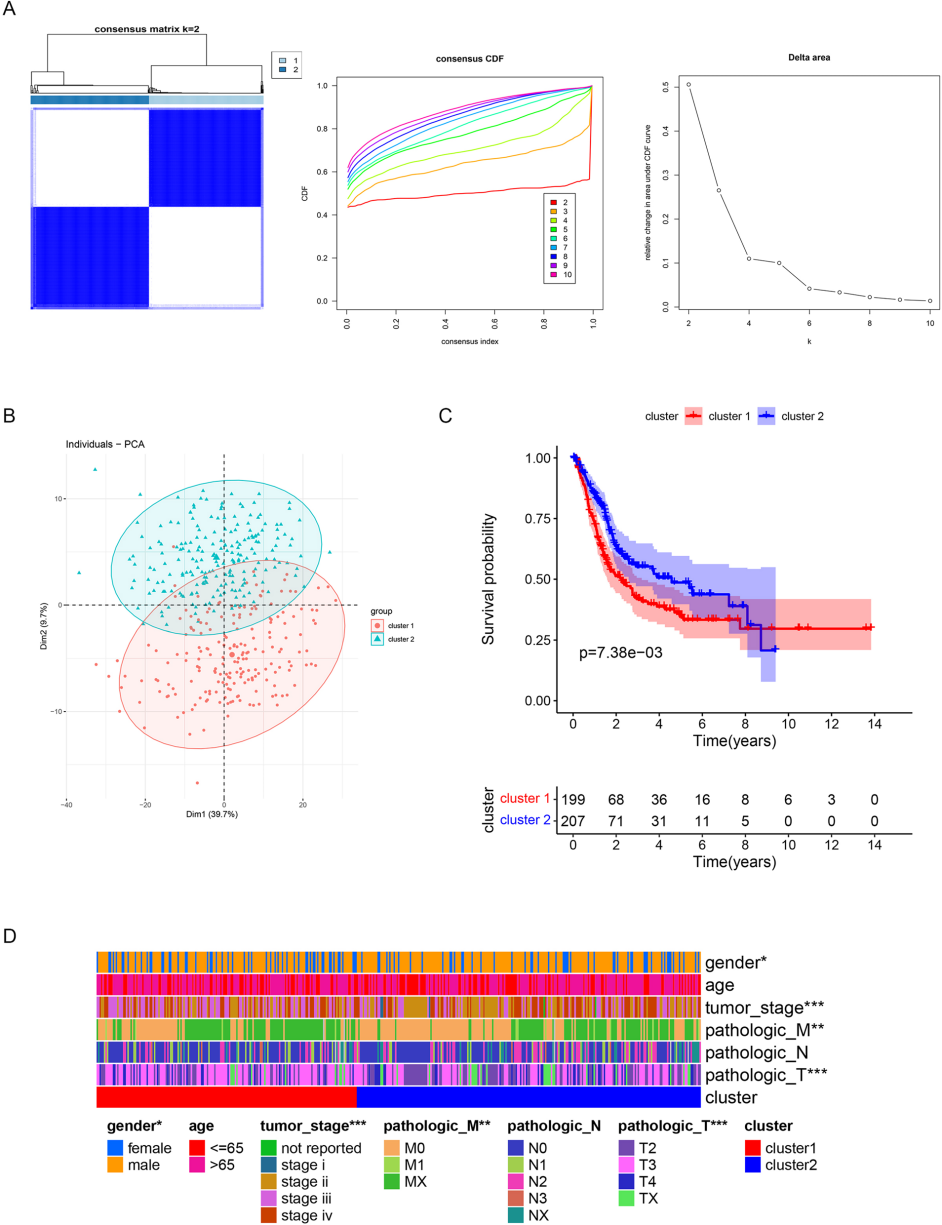
Survival differences existed between patients in cluster 1 and 2. **A**, **B** Results of consistent cluster analysis used to divide BLCA patients into two subtypes. **C** KM curves of the two subtypes (p = 0.00738). **D** Clinical features of the two subtypes. **P < 0.01, ***P < 0.001

Firstly, the expression of m6A methylation regulatory-related and glucose metabolism-related genes was retrieved in the TCGA-BLCA dataset. A total of 326 m6A-related glycolytic genes were identified by Spearman correlation analysis with |Cor|> 0.5 and p < 0.05 as the threshold of significance in BLCA samples (Additional file 1: Table S1), of which 70 were prognosis-related m6A glucose metabolism genes, as identified by univariate Cox analysis. Among these, IGF2BP3, IGF2BP2, SLC16A1, DSC2, TGFBI, P4HA2, ANXA1, and CALU were negative prognostic factors, and CDK10 was the most positive prognostic factor (Additional file 4: Table S4). Based on the expression pattern of prognosis-related m6A glucose metabolism genes, BLCA were divided into two subtypes by consensus clustering analysis (Fig. 1A, B).

The KM curves indicated a significant difference in survival between the two subtypes of patients (p = 0.00738); cluster 2 patients had a better prognosis than that of cluster 1 patients (Fig. 1C). In addition, four clinical features, including gender, pathological T, M, and tumor stage, were significantly different between subtypes (Fig. 1D).

### 34 target genes were associated with metabolism-related pathways in BLCA

**Fig. 2.**
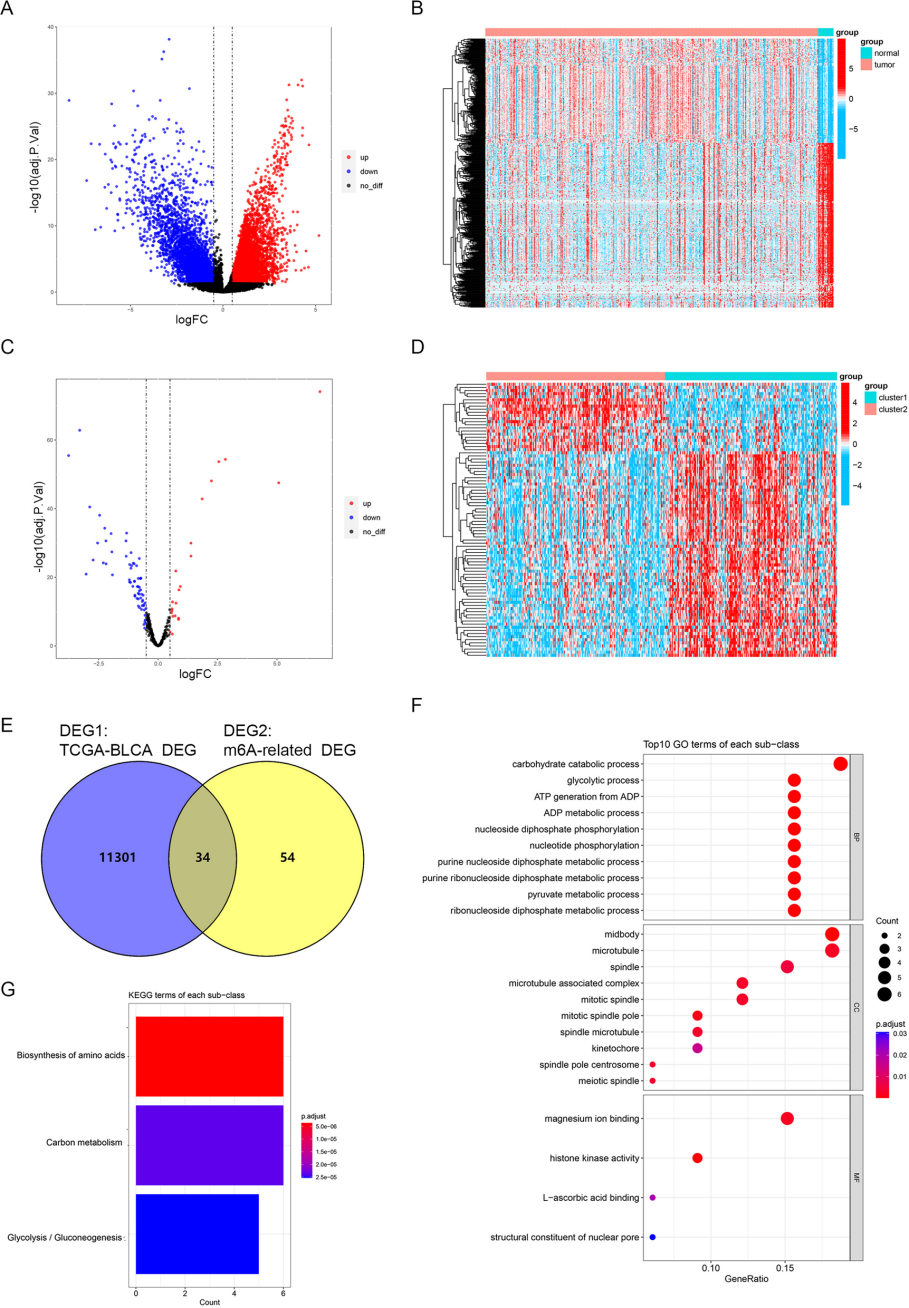
34 target genes were associated with metabolism-related pathways in BLCA.** A**, **B** DEGs between 411 BLCA and 19 HC samples. **C**, **D** DEGs between cluster 1 and cluster 2. **E** Screening of m6A-related glycolytic genes by intersecting DEG1 and DEG2. **F** GO analysis of m6A-related glycolytic genes. **G** KEGG analysis of m6A-related glycolytic genes

There were 7119 upregulated and 4,216 downregulated genes between 411 BLCA and 19 HC samples (Fig. 2A, B), and 22 upregulated and 66 downregulated genes between cluster 1 and cluster 2 samples (Fig. 2C, D). Subsequently, a total of 34 genes associated with BLCA occurrence and development (target genes) were screened by intersecting the differential expressed genes (DEG)1 between BLCA and HC samples and DEG2 between cluster 1and 2 (Fig. 2E, Additional file 5: Table S5).

GO functional analysis showed that the 34 target genes were related to several terms, including carbohydrate catabolism, glycolysis, ATP metabolism, and pyruvate metabolism (Fig. 2F, Additional file 6: Table S6). As for KEGG pathways, these genes were significantly associated with amino acid biosynthesis, carbon metabolism, glycolysis, and gluconeogenesis (Fig. 2G, Additional file 7: Table S7).

### The survival risk model could be used to predict the prognosis of BLCA patients

**Fig. 3:**
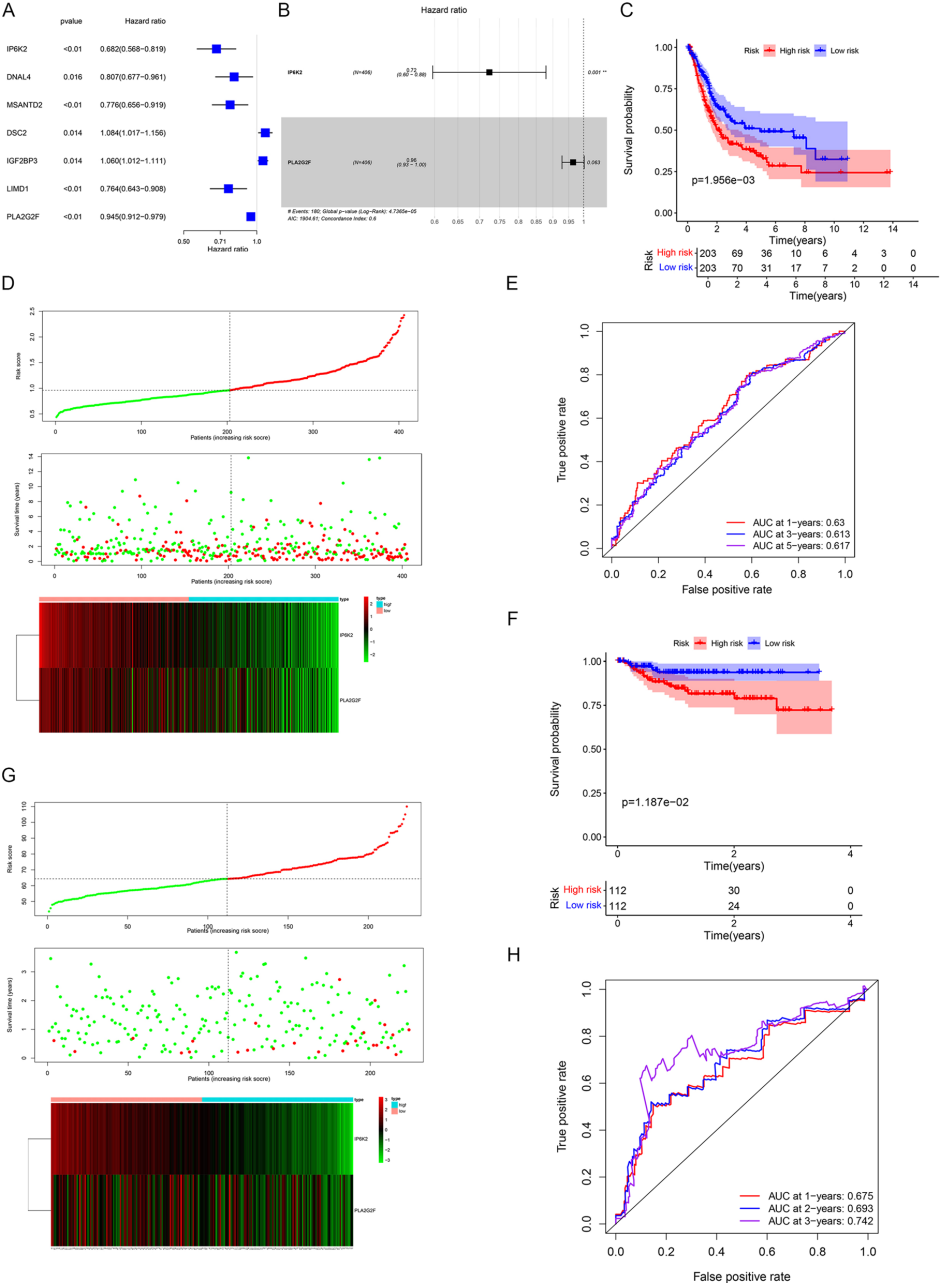
The survival risk model could be used to predict the prognosis of BLCA patients. **A**, **B** Results of Cox analysis identifying IP6K2 and PLA2G2F as positive factors (Hazard Ratio < 1). **C**, **D** Significant survival differences observed between the high- and low-risk groups. **E** ROC curve revealing the potential use of the survival risk model as a prognostic model. **F**–**H** Results of KM, risk and ROC curves in the validation cohort, consistent with the training cohort

Firstly, 34 intersection genes were submitted to univariate Cox regression analysis, and seven genes were obtained with p-value < 0.05 (Fig. 3A). Then, stepwise multivariate Cox regression analysis was used to identify two biomarkers (IP6K2 and PLA2G2F), both of which were positive factors (Hazard Ratio [HR] < 1) (Fig. 3B). The risk score was calculated for each patient, and the median risk score (0.961) was used as the cutoff to divide patients (n = 406, with complete survival data) into the high-risk group (n = 203) and low-risk group (n = 203). The results of KM and risk curves showed significant survival differences between the two groups (Fig. 3C, D). The AUC value of the ROC curve at 1, 3, and 5 years was all greater than 0.6, indicating that the survival risk model could be used as a prognostic model effectively (Fig. 3E). In addition, a significant negative correlation was found between disease risk and expression of IP6K2 and PLA2G2F.

GSE32894 was used as a validation dataset to verify applicability of the model. The results of the KM, risk, and ROC curves were consistent with those of the training dataset (Fig. 3F–H).

### Risk score was an independent risk factor for BLCA patients

**Fig. 4:**
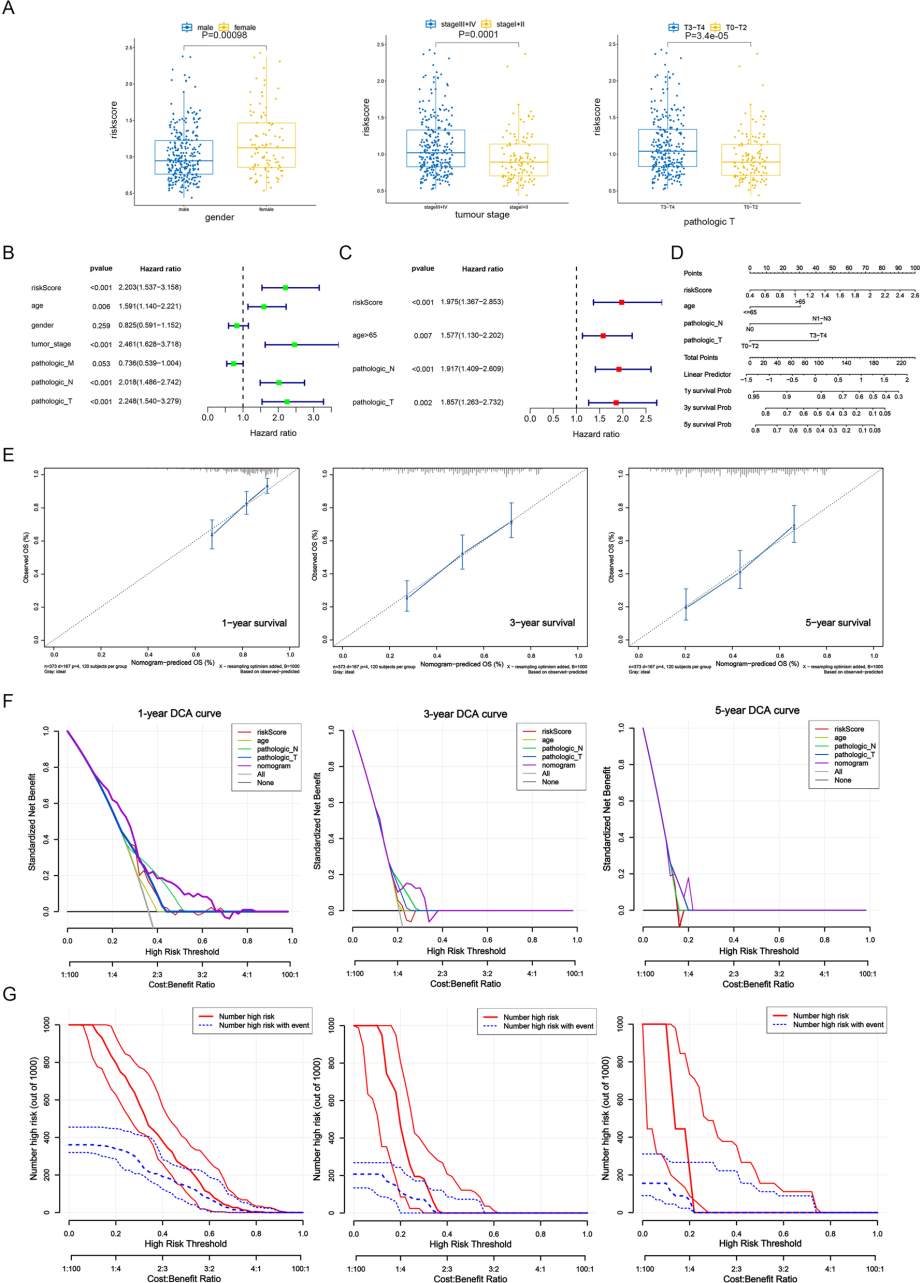
Risk score was an independent risk factor for BLCA patients. **A** Gender, tumor stage, and pathologic T were associated with risk score. **B**, **C** Age, pathology T, N, and risk score were negatively associated with patient survival. **D**, **E** The nomogram for predicting survival in BLCA patients (1-, 3- and 5-year). **F**, **G** Evaluation of the accuracy of prediction using the DCA curve (**F**) and Clinical Impact Curve (**G**). T (Tumor) refers to the extent of the primary tumor; N (Node) refers to the involvement of regional lymph nodes; M (Metastasis) indicates the presence of metastasis

Univariate and multivariate Cox regression analyses were conducted to evaluate the clinical parameters and risk score to assess their prognostic value. The results revealed that gender, tumor stage, and pathological T stage were associated with risk score (Fig. 4A). Four clinical factors, including age, pathology T, N, and risk score, were significantly associated with patient survival (Fig. 4B, C). A nomogram based on these clinical factors was constructed to predict the patients’ 1-, 3-, and 5-year survival rates; the calibration curve showed that the slopes were closest to 1 (Fig. 4D, E). In addition, the nomogram model had a higher benefit rate than those of others, and the “number high risk” curves coincided with “number high risk with event” curves. These results indicated that the nomogram model has accurate predictive ability for BLCA (Fig. 4F, G).

### Difference in function enrichment between the high- and low-risk groups

**Fig. 5:**
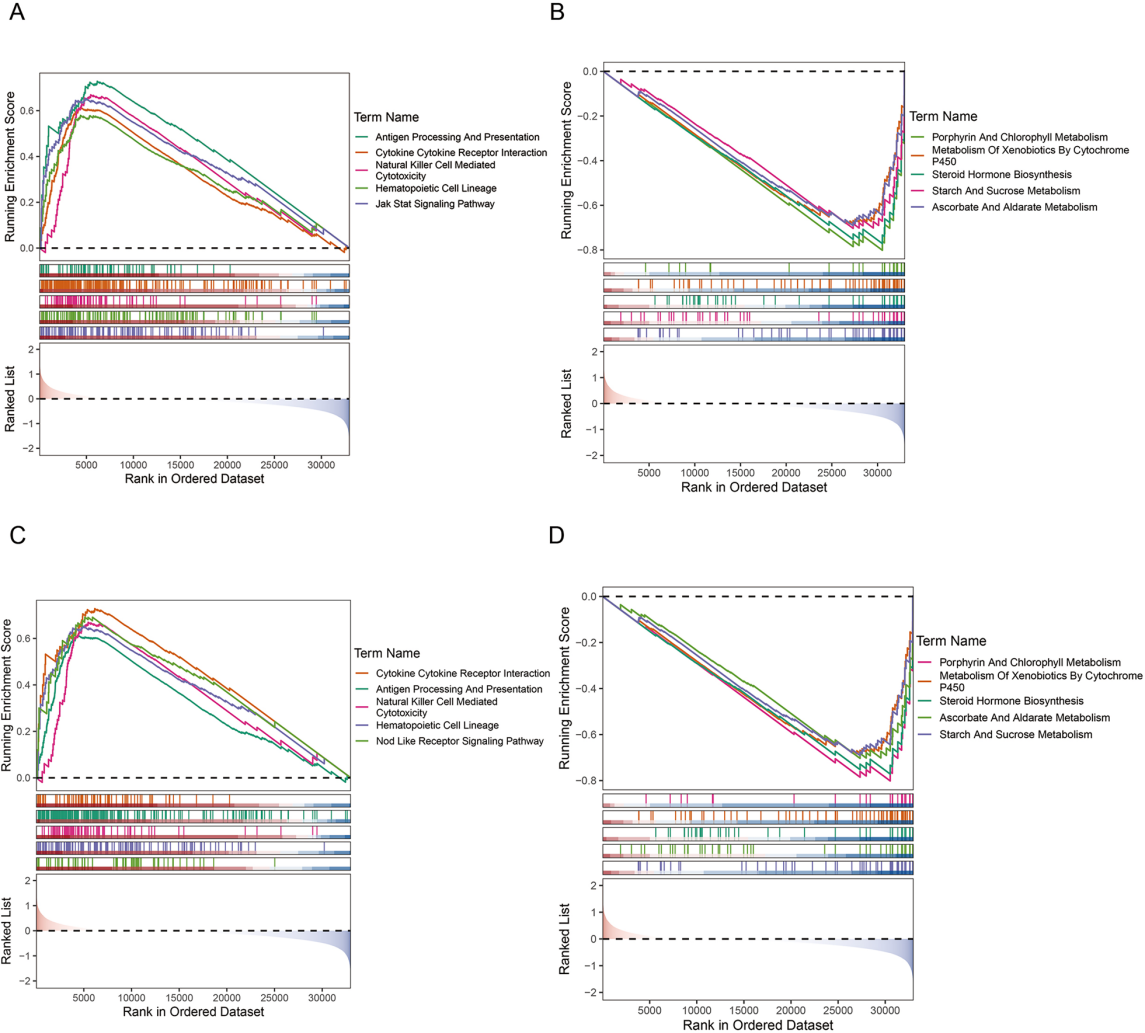
The difference in function enrichment between the high- and low-risk groups **A** GO analysis showing enrichment of T-cell activation, antigen receptor-mediated signaling pathway, B-cell differentiation, B-cell mediated immunity, and B-cell receptor signaling pathway in the high-risk groups. **B** GO analysis showing enrichment of cellular glucuronidation, estrogen metabolism, miRNA-mediated gene silencing by inhibition of translation, sodium ion homeostasis, and translation repressor activity in the low-risk groups. **C** KEGG analysis showing enrichment of antigen processing and presentation and of CAMs in the high-risk groups. **D** KEGG analysis showing enrichment of ascorbate and aldarate metabolism, drug metabolism, and of other enzymes, in the low-risk groups

To investigate the potential molecular mechanisms of risk model, we performed GSEA enrichment analysis between the high- and low-risk groups. The GSEA results for the two groups are shown in Fig. 5A–D. T-cell activation, antigen receptor-mediated signaling pathway, B-cell differentiation, B-cell-mediated immunity, and B-cell receptor signaling pathway were enriched in the high-risk groups (Fig. 5A), whereas cellular glucuronidation, estrogen metabolism, miRNA-mediated gene silencing by inhibition of translation, sodium ion homeostasis, and translation repressor activity were enriched in the low-risk groups (Fig. 5B). The genes expressed in the high-risk groups were involved in antigen processing and presentation, cell adhesion molecules (CAMs), chemokine signaling, hematopoietic cell lineage, and JAK/STAT signaling (Fig. 5C). Meanwhile, genes expressed in the low-risk groups were involved in ascorbate and aldarate metabolism, drug metabolism, other enzymes, metabolism of xenobiotics by cytochrome p450, porphyrin and chlorophyll metabolism, and steroid hormone biosynthesis (Fig. 5D). The GSEA results revealed potential pathways or mechanisms that were activated during tumorigenesis and development, which can help us evaluate the prognosis of BLCA.

### Significant differences were observed in immune microenvironment between the two risk groups

**Fig. 6:**
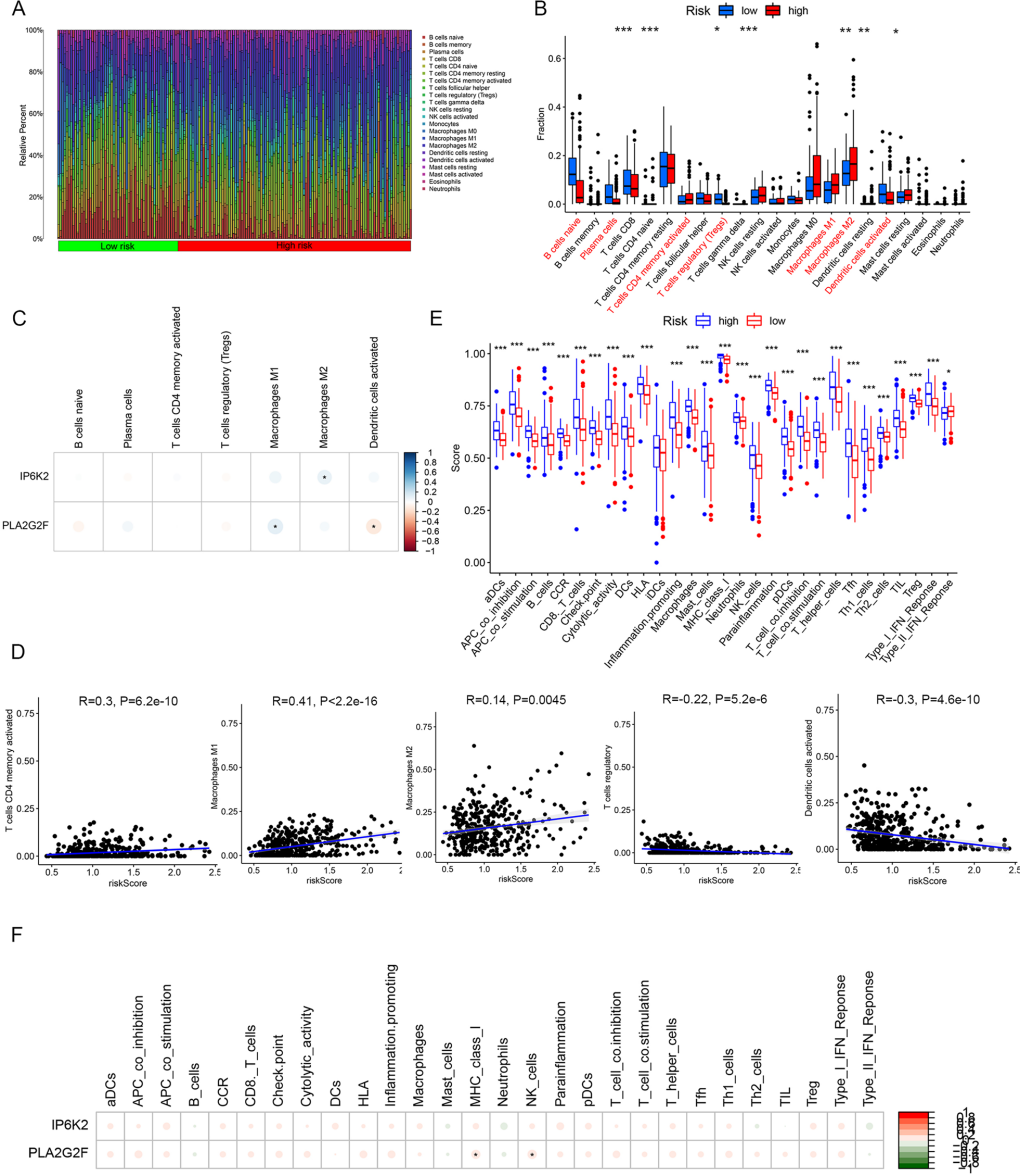
Significant differences were observed in immune microenvironment between the two risk groups. **A**, **B** Seven differential immune cells were identified in the high- and low-risk groups. **C** Heat map of correlation between biomarkers and differential immune cells. **D** Correlation between risk scores and differential immune cells. **E** Box plot of differential immune functions between the high- and low-risk groups. **F** Heat map of correlation between biomarkers and differential immune function. *P < 0.05, **P < 0.01, ***P < 0.001, ****P < 0.0001

The CIBERSORT algorithm was used to investigate the differences in immune cell infiltration between the high- and low-risk groups. Seven immune cells, including naive B cells, plasma cells, activated memory CD4 + T cells, regulatory T cells, M1 macrophages, M2 macrophages, and activated dendritic cells, were found to have significantly different expression levels in the high- and low-risk groups. Among these, the immune infiltration of activated memory CD4 + T cells, M1 and M2 macrophages was significantly higher in the high-risk groups (Fig. 6A, B). IP6K2 expression correlated positively with M2 macrophages, while PLA2G2F expression correlated positively with M2 macrophages and negatively with activated dendritic cells (Fig. 6C). Meanwhile, risk score correlated positively with activated memory CD4 + T cells, M1 and M2 macrophages, and negatively with regulatory T cells and activated dendritic cells (Fig. 6D). In addition, 28 immune functions were significantly different between the high- and low-risk groups; among these, IP6K2 expression correlated positively with MHC class 1 and NK cell function (Fig. 6E, F). In summary, we speculated that immune microenvironment was associated with the occurrence and development of BLCA.

### The risk score can predict the effectiveness of immunotherapy

**Fig. 7:**
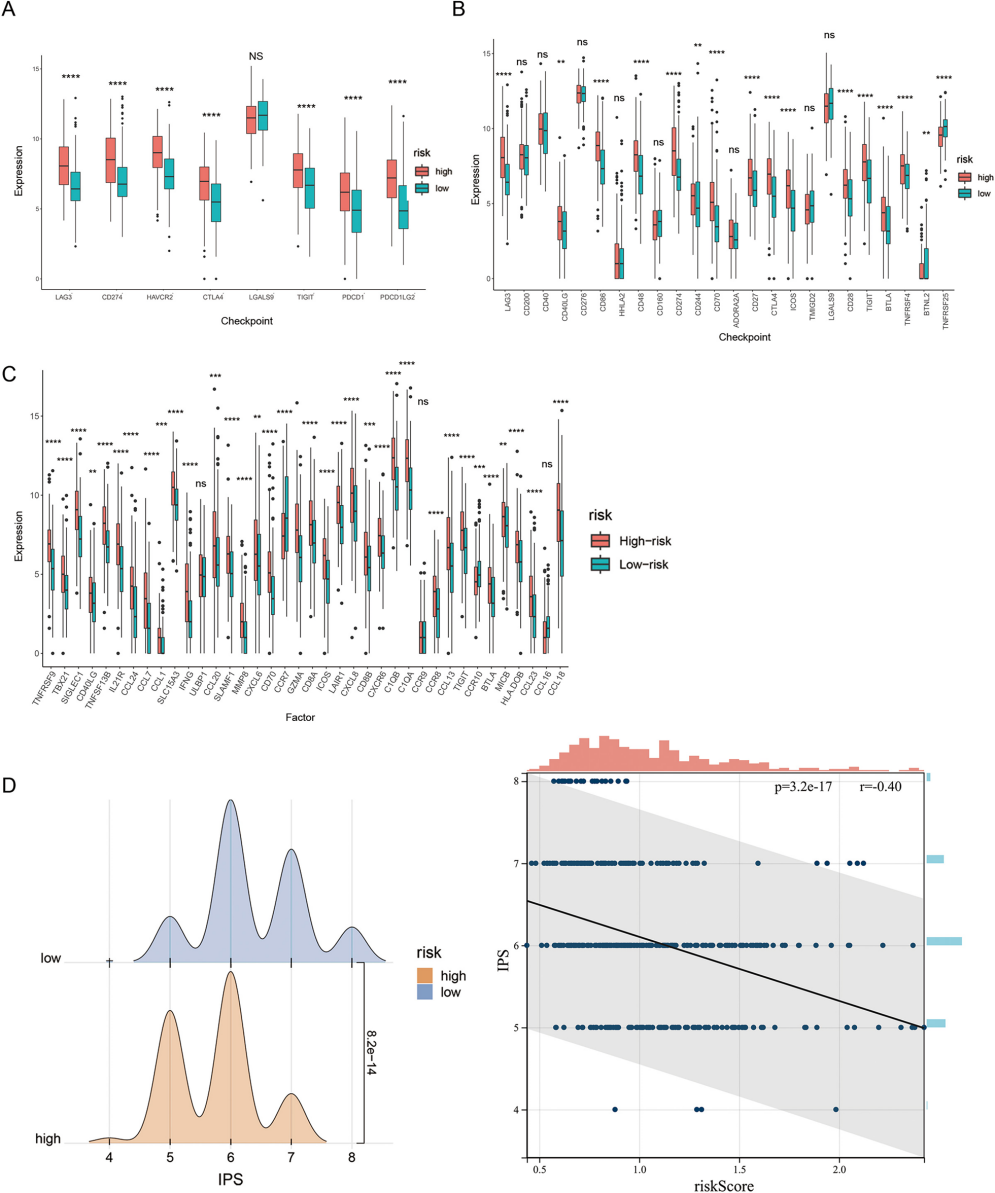
The risk score can predict the effectiveness of immunotherapy. **A**–**C** Expression of seven immune checkpoints, 14 ICIs and 32 immune factors in the high- and low-risk groups. **D** IPS scores in the high- and low-risk groups. *P < 0.05, **P < 0.01, ***P < 0.001, ****P < 0.0001

As immunotherapy became a promising strategy for the treatment of tumors, we evaluated differences in immunotherapy response between high- and low-risk groups. The expression levels of seven immune checkpoints (CD274, CTLA-4, LAG-3, HAVCR2, PDCD1, PDCD1LG2 and TIGIT), 14 immune checkpoint inhibitors (LAG3, CD40LG, CD86, CD48, CD274, CD244, CD70, CD27, CTLA4, ICOS, CD28, TIGIT, BTLA, TNFRSF4) and 32 immune factors were significantly higher in the high-risk groups, while those of two immune checkpoint inhibitors (BTNL2 and TNFRSF25) and three immune factors (CCR7, CCR10, CCL18) were significantly higher in the low-risk groups (Fig. 7A–C). Furthermore, the risk score significantly negatively correlated with the IPS score (Fig. 7D), indicating that patients in the low-risk group were more likely to benefit from immunotherapy.

### The risk score model can predict the drug sensitivity between high- and low-risk groups

Considering that the risk score model could be used to predict the prognosis of BLCA patients, we further investigated the relationship between risk score and chemotherapy resistance. The results revealed that there were significant differences in the IC50 values of 114 drugs between the high- and low-risk groups. (Additional file 8: Table S8), and we further analyze the differences in drug sensitivity between the high- and low-risk groups for 10 drugs, such as parthenolide, sunitinib, cyclopamine, bexarotene, etc. (Additional file 9: Fig. S9). Therefore, we believe that this risk score model can be utilized to predict the sensitivity of BLCA patients to chemotherapy drugs, which could improve the effectiveness of chemotherapy.

### IP6K2 and PLA2G2F were downregulated in bladder cancer tissues and could regulate bladder cancer cell proliferation in vitro

**Fig. 8:**
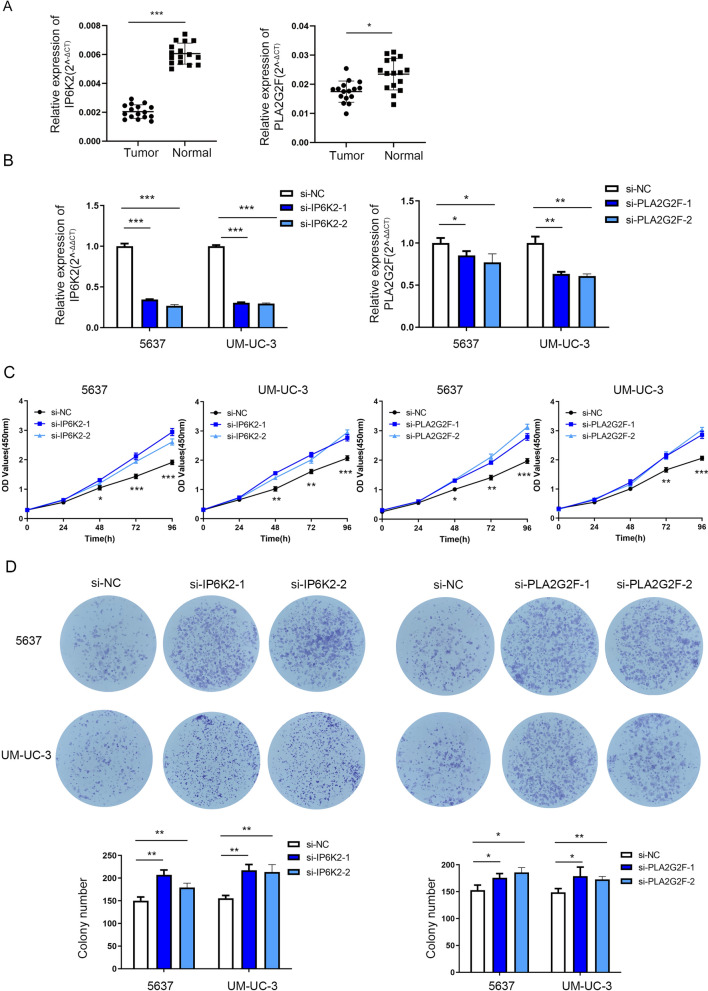
IP6K2 and PLA2G2F were downregulated in bladder cancer tissues and could regulate bladder cancer cell proliferation in vitro. **A** IP6K2 and PLA2G2F expression in bladder cancer and matched adjacent normal tissues detected by RT-qPCR. **B** Efficiency of IP6K2 and PLA2G2F knockdown verified by RT-qPCR. **C** The proliferative ability of IP6K2 and PLA2G2F knockdown cells was determined using CCK-8 assay. **D** The colony-forming ability of IP6K2 or PLA2G2F knockdown cells was determined using colony formation assay. *P < 0.05, **P < 0.01, ***P < 0.001

IP6K2 and PLA2G2F expression in bladder cancer and adjacent tissues was detected using RT-qPCR. Expression levels of IP6K2 and PLA2G2F were significantly downregulated in cancer tissues compared with their expression levels in adjacent tissues (Fig. 8A).

Effect of IP6K2 and PLA2G2F on cell proliferation in vitro was explored to determine their role in bladder cancer cell proliferation. Efficiency of IP6K2 and PLA2G2F knockdown was verified using RT-qPCR (Fig. 8B). CCK-8 assays demonstrated that IP6K2 knockdown significantly promoted cancer cell proliferation in 5637 and UM-UC-3 cells (Fig. 8C). Moreover, the colony-forming ability of 5637 and UM-UC-3 cells enhanced after IP6K2 or PLA2G2F knockdown (Fig. 8D).

## Discussion

As bladder cancer has a high risk of recurrence after treatment, it is important to explore its molecular mechanisms and discover novel therapeutic targets. Aberrant regulation of m6A modification, a hotspot in cancer research, is closely associated with tumorigenesis and metastasis [5]. Dysregulation of m6A modification may impact the expression of genes related to tumor progression, some of which are associated with glycolysis [6, 20, 21]. The relationship between m6A modification and glycolysis in tumors is an area of active research and has important implications in understanding the molecular mechanisms underlying tumorigenesis and cancer progression. m6A modification can influence the expression of glycolysis-related genes by regulating their mRNAs’ stability and translation [6, 20]. This can activate the glycolytic pathway in tumor cells, thereby providing more energy to meet their rapid growth and proliferation demands. Moreover, tumor cells often rely on glycolysis for their energy needs, and this can create a local environment with increased lactate production and decreased pH value. This acidic microenvironment can influence immune cell function and suppress immune responses, making it easier for tumor cells to evade the surveillance and elimination of immune system [22, 23]. In this study, we identified a group of glucose metabolism genes associated with m6A regulator-related genes, aiming to further explore their roles in the occurrence and development of bladder cancer.

We identified IP6K2 and PLA2G2F as BLCA biomarkers using Cox regression analysis. Two risk groups with different prognoses were demonstrated, which may help develop different therapeutic strategies. The survival risk model was verified using a ROC curve, which could be used to predict the prognosis of BLCA patients. Moreover, the analysis results of validation datasets were consistent with those of the training dataset, suggesting a reliable and effective survival risk model.

IP6K2 converts InsP6 into InsP7/PP-InsP5 [24], all of which are important members of the IPK family. Additionally, it mediates apoptosis by facilitating the DNA damage-induced cell death pathway [25, 26]. We found that IP6K2 knockdown promotes bladder cancer cell proliferation and colony formation. Aberrant PLA2G2F expression is associated with several malignancies [27, 28]. In addition, PLA2G2F knockdown also promotes proliferation and colony formation of bladder cancer cells.

Aerobic glycolysis provides energy for tumor proliferation; in addition, it generates various tumor metabolites that reshape the tumor microenvironment (TME). Specifically, tumor aerobic glycolysis can lead to nutrient deprivation in the microenvironment and induce hypoxia with extracellular lactic acid accumulation, thus forming an inhibitory immune microenvironment [8]. The TME consists of dynamically balanced tumor cells, immune-infiltrating cells, fibroblasts, and cytokines. Tumor-infiltrating immune cells (TIICs) play important roles in tumor growth, invasion, and metastasis, making them effective targets for immunotherapy [29, 30]. TIICs are closely related to clinical outcomes of BLCA and can serve as effective targets for the development of new immunotherapies for BLCA patients [31–33]. To explore immune cell infiltration in the high- and low-risk groups, we conducted immune cell infiltration analysis, using the CIBERSORT algorithm to obtain the infiltration of each type of immune cell. Significant differences in the infiltration of 28 immune functions were found between the high- and low-risk groups. Among them, IP6K2 was significantly positively correlated with MHC class 1 and NK cell function.

After analyzing the expression of immune checkpoints in the high- and low-risk groups, we found significant upregulation of 7 immune checkpoints, 14 ICIs, and 32 immune factors in the high-risk groups, and of two ICIs and three immune factors in the low-risk groups. Moreover, IPS was significantly higher in the low-risk groups, indicating greater sensitivity of these patients to immunotherapy. Immune checkpoint receptors are located on the surface of immune cells. After binding to ligands, they play a role in regulate immune response and maintain self-tolerance [34]. Cancer cells use this feature to evade immune system surveillance, leading to immune escape [35, 36]. Immune checkpoint inhibitors (ICIs) are a novel class of cancer therapeutic drugs that enhance the anti-tumor response of the immune system by inhibiting immune escape of tumor cells. By altering the m6A modification of specific genes, the expression of tumor-associated antigens can be up-regulated, thereby enhancing the anti-tumor immune response [37, 38]. Most tumor cells exhibit enhanced glycolytic capacity, which enables them to survive in hypoxic and nutrient-poor microenvironment and provides energy for rapid growth. This process leads to the production of large amounts of lactate and other metabolic byproducts, which can inhibit the function of immune cells [39]. One of the goals of immunotherapy is to reverse the suppressive effect of tumor cells on immune cells by interfering with their glucose metabolism. ICIs eliminate tumor cells by restoring the activity of T cells. These drugs can alter the metabolism of T cells, increasing their demand for glucose in order to enhance their tumor killing activity [39]. Simultaneously, it is necessary to reduce the inhibitory effect on the immune system by intervening in the process of tumor cells’ glucose metabolism [40, 41]. Therefore, it is crucial to fully understand and utilize these relationships in order to optimize immunotherapy strategies and improve the effectiveness of immunotherapy.

## Conclusions

In conclusion, our study identified two biomarkers (IP6K2 and PLA2G2F) associated with BLCA prognosis. We constructed a risk model which could effectively predict patient prognosis and immunotherapy response and guide individualized immunotherapy. However, further validation using basic experiments is required to confirm our findings.

### Supplementary Information

**Additional file 1: Table S1.** Glycolysis-related genes and m6a-related genes.

**Additional file 2: Table S2.** siRNA sequences used in this study.

**Additional file 3: Table S3.** Primes sequences for RT-qPCR used in this study.

**Additional file 4: Table S4.** Univariate Cox analysis of m6A-related glycolytic genes

**Additional file 5: Table S5.** m6A-related glycolytic genes

**Additional file 6: Table S6.** GO analysis of m6A-related glycolytic genes.

**Additional file 7: Table S7.** KEGG analysis of m6A-related glycolytic genes

**Additional file 8: Table S8.** Sensitivity analyses of 138 drugs in patients between high- and low-risk groups

**Additional file 9: Figure S1.** Differences in drug sensitivity between high- and low-risk groups.

## Data Availability

The datasets used and/or analysed during the current study are available from the corresponding author on reasonable request.

## Abbreviations

BLCA: Bladder urothelial carcinoma
m6A: N6-methyladenine
TME: Tumor microenvironment
TCGA: The Cancer Genome Atlas
GEO: Gene Expression Omnibus
K-M curves: Kaplan–Meier (K–M) curves
GO: Gene Ontology
KEGG: Kyoto Encyclopedia of Genes and Genomes
ROC: Receiver operating characteristic
DCA: Decision curve analysis
GSEA: Gene set enrichment analysis
IPS: Immune phenotype scores
IC50: Half-maximal inhibitory concentration
CCK-8: Cell counting kit-8
DEGs: Differentially expressed genes
TIICs: Tumor-infiltrating immune cells
ICIs: Immune checkpoint inhibitors
