# Supplementary material for: Identifying prognostic characteristics of m6A-related glycolysis gene and predicting the immune infiltration landscape in bladder cancer
Source: Cancer Cell Int. 2023 Nov 28;23:300. doi: 10.1186/s12935-023-03160-w (PMC10683108; doi:10.1186/s12935-023-03160-w)
Supplement: Supplementary file 2 — Additional file 2: Table S2. siRNA sequences used in this study. [file 12935_2023_3160_MOESM2_ESM.docx]

**Supplementary Table 2. siRNA sequences used in this study.**

| Gene | Sequences (5’-3’) |
| --- | --- |
| si-IP6K2-1 | sense: GCACCGUAAAGAGGAGAAATT |
|  | antisense: UUUCUCCUCUUUACGGUGCTT |
| si-IP6K2-2 | sense: GGCAGCUCAUGUUCAUGAATT |
|  | antisense: UUCAUGAACAUGAGCUGCCTT |
| si-PLA2G2F-1 | sense: GCUGGAUAGAUGCUUCUCUTT |
|  | antisense: AGAGAAGCAUCUAUCCAGCTT |
| si-PLA2G2F-2 | sense: GCAUGUGUGACAAGAACAUTT |
|  | antisense: AUGUUCUUGUCACACAUGCTT |
| si-NC | sense: UUCUCCGAACGUGUCACGUTT |
|  | antisense: ACGUGACACGUUCGGAGAATT |
