# Supplementary material for: Identifying prognostic characteristics of m6A-related glycolysis gene and predicting the immune infiltration landscape in bladder cancer
Source: Cancer Cell Int. 2023 Nov 28;23:300. doi: 10.1186/s12935-023-03160-w (PMC10683108; doi:10.1186/s12935-023-03160-w)
Supplement: Supplementary file 3 — Additional file 3: Table S3. Primes sequences for RT-qPCR used in this study. [file 12935_2023_3160_MOESM3_ESM.docx]

**Supplementary Table 3. Primes sequences for RT-qPCR used in this study.**

| **Gene** | **Sequences (5’-3’)** |
| --- | --- |
| IP6K2-forward | GTCACCAGCAACAGTTACAGAGAA |
| IP6K2-reverse | TTGTCGTGTGCCCATCTTGAG |
| PLA2G2F-forward | CTGCTGCTACCAGGAACTCTT |
| PLA2G2F-reverse | CACTCTGTCTTGTTGAGGTCACT |
| β-actin-forward | GGCGGCACCACCATGTACCCT |
| β-actin -reverse | AGGGGCCGGACTCGTCATACT |
