# Supplementary figures and images for: Identifying prognostic characteristics of m6A-related glycolysis gene and predicting the immune infiltration landscape in bladder cancer
Source: Cancer Cell Int. 2023 Nov 28;23:300. doi: 10.1186/s12935-023-03160-w (PMC10683108; doi:10.1186/s12935-023-03160-w)

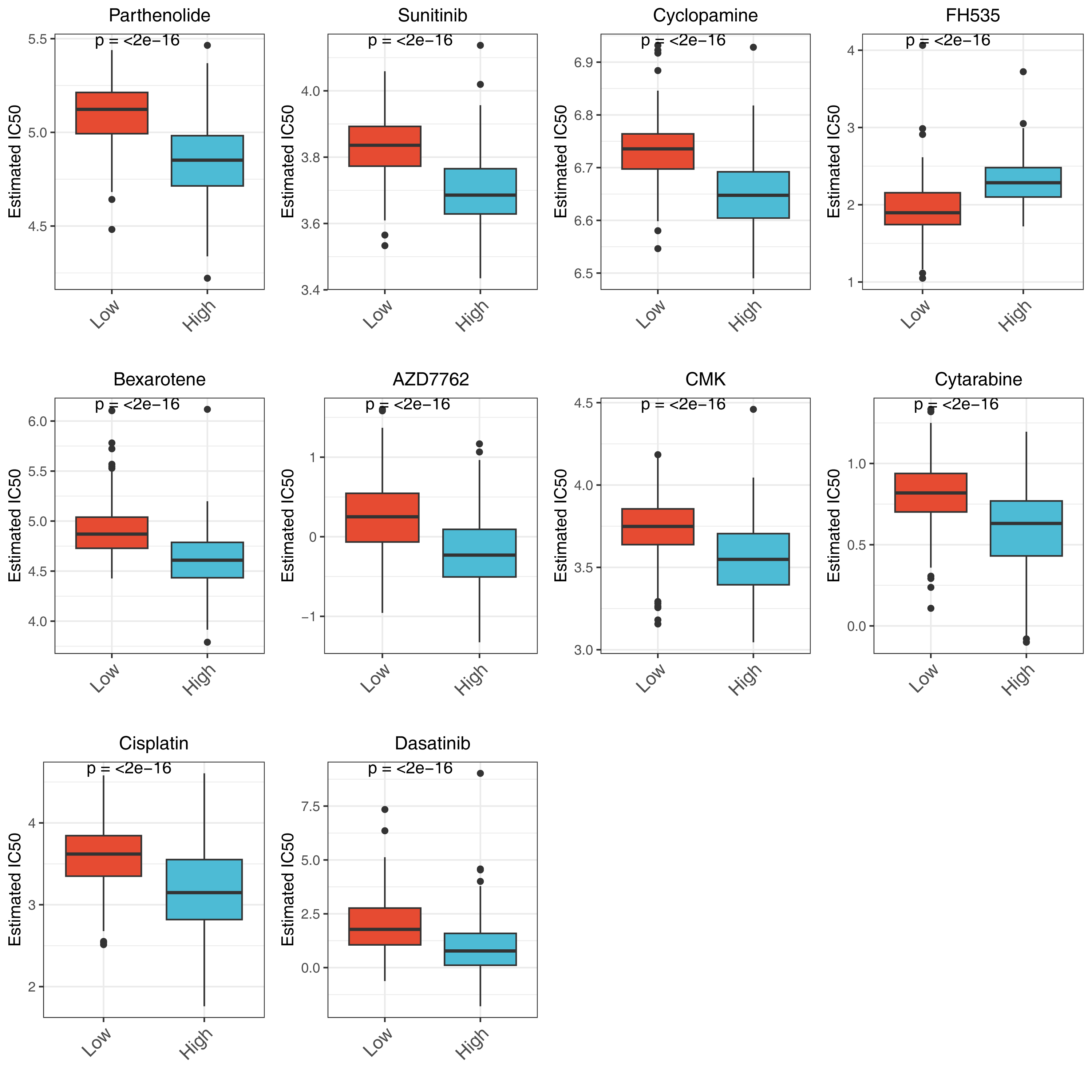

Supplement: Supplementary file 9 — Additional file 9: Figure S1. Differences in drug sensitivity between high- and low-risk groups. [file 12935_2023_3160_MOESM9_ESM.tif]
